# Towards the Construction of Expressed Proteomes Using a *Leishmania tarentolae* Based Cell-Free Expression System

**DOI:** 10.1371/journal.pone.0014388

**Published:** 2010-12-21

**Authors:** Oleksiy Kovtun, Sergey Mureev, Wayne Johnston, Kirill Alexandrov

**Affiliations:** Institute for Molecular Bioscience, The University of Queensland, Brisbane, Queensland, Australia; Victor Chang Cardiac Research Institute, Australia

## Abstract

The adaptation of organisms to a parasitic life style is often accompanied by the emergence of novel biochemical pathways absent in free-living organisms. As a result, the genomes of specialized parasitic organisms often code for a large number (>50%) of proteins with no detectable homology or predictable function. Although understanding the biochemical properties of these proteins and their roles in parasite biogenesis is the next challenge of molecular parasitology, analysis tools developed for free-living organisms are often inadequate for this purpose. Here we attempt to solve some of these problems by developing a methodology for the rapid production of expressed proteomes in cell-free systems based on parasitic organisms. To do so we take advantage of Species Independent Translational Sequences (SITS), which can efficiently mediate translation initiation in any organism. Using these sequences we developed a single-tube *in vitro* translation system based on the parasitic protozoan *Leishmania tarentolae*. We demonstrate that the system can be primed directly with SITS containing templates constructed by overlap extension PCR. To test the systems we simultaneously amplified 31 of *L. tarentolae's* putative translation initiation factors and phosphatases directly from the genomic DNA and subjected them to expression, purification and activity analysis. All of the amplified products produced soluble recombinant proteins, and putative phosphatases could be purified to at least 50% purity in one step. We further compared the ability of *L. tarentolae* and *E. coli* based cell-free systems to express a set of mammalian, *L. tarentolae* and *Plasmodium falciparum* Rab GTPases in functional form. We demonstrate that the *L. tarentolae* cell-free system consistently produced higher quality proteins than *E. coli*-based system. The differences were particularly pronounced in the case of open reading frames derived from *P. falciparum*. The implications of these developments are discussed.

## Introduction

The evolutionary pressures exerted on parasitic organisms differ significantly from that experienced by free living organisms, resulting in the emergence of unique biochemical activities and regulatory mechanisms. Recent advances in DNA technologies have allowed the sequencing of numerous parasitic species, ranging from viruses to multicellular organisms. Analysis of genomes from protozoan parasites has revealed that in some cases more than half of their coding genome encode proteins with no obvious homologies and therefore predictable function [1]. Understanding the biochemical properties of these proteins and their roles in parasite biogenesis is the next biggest challenge of molecular parasitology. Progress in this direction is often hampered by the fact that with the exception of virus-based systems, most molecular biology tools were derived from or developed for a small subset of autonomous model organisms. Attempts to use these systems to express and reconstitute parasitic proteomes are often unsuccessful due to evolutionary divergence of the gene transcription and translation apparatus. Although these problems are not limited to parasitic organisms, they provide a significant impediment to the biochemical and structural analysis of proteomes from prominent protozoan parasites such as *Plasmodium spp.*, and to a lesser extent *Trypanosoma* and *Leishmania spp.* [2], [3].

**Figure 1:**
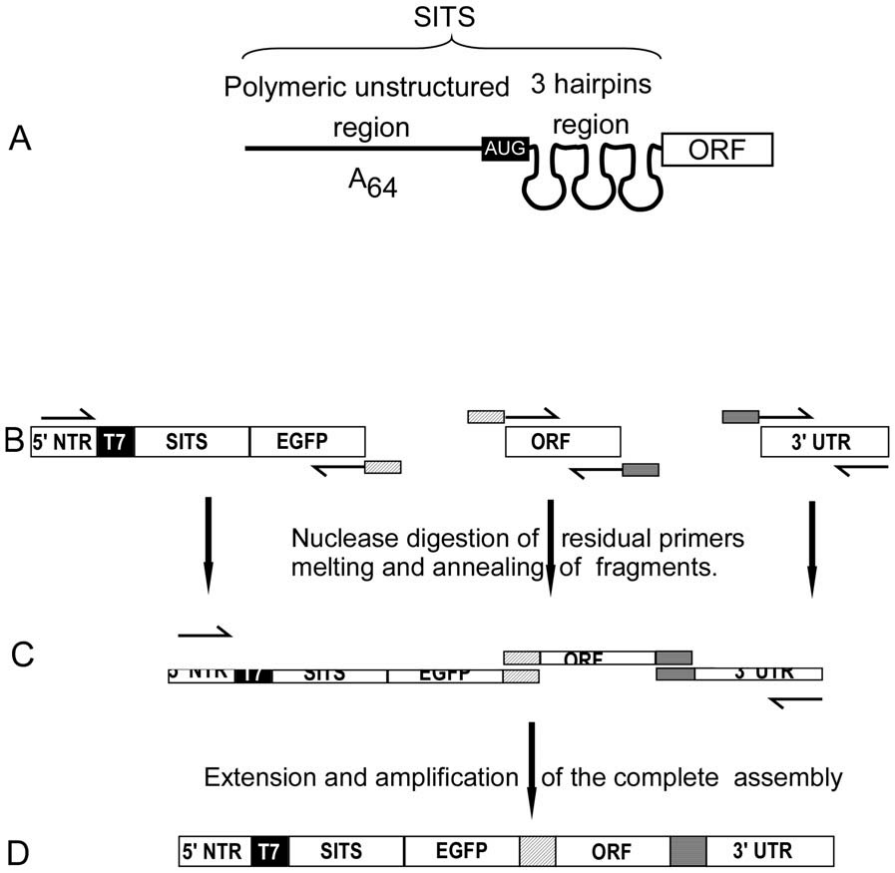
Use of Species-Independent Translational Sequence (SITS) in Overlap Extension (OE) PCR-mediated assembly of templates for *in vitro* translation. (A) Schematic representation of SITS structure. (B) Scheme of purification-free OE-PCR for synthesis of DNA templates for cell-free translation. PCR amplification of individual fragments with partially overlapping sequences. 5′ NTR – 5′ not transcribed regions, 3′ UTR – 3′ untranslated region. (C) Removal of residual primers from PCR reaction by exonuclease I treatment. (D) Fragments are fused and amplified by Overlap Extension PCR in the presence of the flanking primers.

Although translation initiation mechanisms greatly diversified in evolution, the basic mechanisms of ribosomal function remained largely conserved. We have previously designed a series of RNA sequences that contain a long unstructured polymeric stretch followed by a sequence forming three short stem hairpins (Fig. 1A) [4]. The hairpins are translated into a short N-terminal extension of the following open reading frame. According to the current model, the unstructured polymeric stretch promotes pre-initiation complex assembly and enables one-dimensional sliding of the small ribosomal subunit in search of the start codon [4], [5], [6], [7]. This is presumed to promote recognition of the start codon by the pre-initiation complex and facilitate recruitment of the large ribosomal subunit. We demonstrated that mRNAs with these translation leaders could mediate productive protein translation in cell extracts from *E. coli*, *Saccharomyces cerevisiae*, insect cells, wheat germ, *L. tarentolae* and rabbit reticulocytes [4]. We therefore designated these synthetic translation leaders Species-Independent Translational Sequences (SITS). Although use of SITS results in a 17 amino acid N-terminal extension, this did not have obvious adverse effects on the activity of the analyzed recombinant proteins [4].

The fact that translation competent lysate could be prepared from a representative of *Leishmania* species encouraged us to test whether this represented an opportunity to produce *Leishmania* proteins in a homologous expression system. If successful this would greatly accelerate the understanding of *Leishmania* and *Trypanosoma* biology on a biochemical and structural level. In particular the structural analysis of parasitic proteomes requires departure from *E. coli* expression systems as evidenced by high attrition rates of structural genomics pipelines [2], [3]. Availability of an efficient protein expression platform for parasitic proteins would enable identification of potential vaccines and drug targets by providing access to recombinant proteins for immunization, biochemical experiments, high throughput screening and protein array construction. Here we demonstrate that the *L. tarentolae* cell-free expression system (LTE) can be used for rapid expression and purification of *Leishmania* proteins using genomic amplicons constructed by overlap extension PCR(OE-PCR). We further demonstrate that the developed technology can be used for *in vitro* expression of the AT rich genes of *Plasmodium falciparum* in active form.

## Results

### Experimental design and technology development

To develop a methodology allowing efficient genome to proteome conversion, several conditions must be fulfilled. Firstly the expression system must support the folding of target proteins and be rapid, efficient, inexpensive and scalable. Secondly the target open reading frames must be efficiently converted into expression templates (preferably without the need for cloning). Finally the resulting recombinant proteins should be purified in a minimal number of steps (preferably one).

As a test example in our study we chose *L. tarentolae* – a lizard parasite extensively used as a model system for *Leishmania* species that infect mammals [8]. We previously described that cell extract from this species (LTE) displays efficient protein translation when primed with mRNAs carrying SITS [4]. In order to adapt the system to multiplexed applications, we supplemented the system with T7 RNA polymerase and rNTPs. This links the transcription and translation processes and allows the system to be primed directly with coding DNAs (see Materials and Methods). As we typically prepare 50 ml of the coupled lysate from 10 liters of *L. tarentolae* culture in a one day procedure, we concluded that the system was sufficiently scalable and inexpensive to be useful in multiplexed analysis [4].

Next we sought a way of departing from cloning-based template preparation methods. Although plasmid based technology has been used for the expression of mammalian proteomes, it requires a large investment as well as laboratory automation [9]. We decided to seek a method that would allow direct amplification and fusion of genomic or cDNA with the SITS required for efficient translational initiation. To this end we employed OE-PCR in which ORFs of interest were fused to regulatory elements [10], [11]. The main shortcoming of this method is the requirement for separation of primer oligonucleotides and primary PCR products by gel purification, limiting the throughput of the procedure. To overcome this bottleneck we decided to remove the primers enzymatically, using nucleases specific for single stranded DNA (Fig. 1B).

To test this approach we firstly assembled templates for a series of arbitrary genes from our laboratory. In these assemblies we included the 5′ SITS sequence and EGFP encoding sequence 3′-fused to the ORF of interest. The latter is advantageous as it allows real time monitoring of protein synthesis in the translation reaction and can be used to isolate the resulting fusion protein by affinity chromatography (see below).

**Figure 2:**
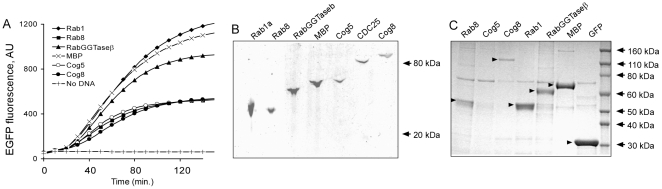
*In vitro* translation of PCR products encoding a set of genes fused to egfp gene. (A) Progression of *in vitro* translation reactions primed with PCR products was monitored by changes in EGFP fluorescence in a 384-well plate. Abbreviations: Rab1 and Rab8 – human small RabGTPases, RabGGTaseβ - β subunit of rat Rab geranylgeranyl transferase, MBP - *E. coli* maltose binding protein, Cog5 – subunit 5 of yeast Conserved Golgi oligomeric complex, Cog8 – subunit 8 of yeast Conserved Golgi oligomeric complex, CDC25 - human tyrosine and threonine phosphatase. (B) Fluorescent scan of SDS-PAGE gel loaded with 2µl of unboiled *in vitro* translation mixtures. The scanning was performed on a Typhoon scanner (GE Healthcare) using 488 nm excitation laser and 520 BP40 emission filter. (C) Coomassie stained SDS-PAGE of eluates from GFP-Cap resin incubated with 150µl of *in vitro* translation reactions. The arrowheads show the position of the recombinant proteins.

Next we tested the ability of the crude OE-PCR mixture to prime the *in vitro* translation reaction. 2 µl of the PCR mixture was added directly to the 18 µl of coupled LTE, and progress of the translation reaction was monitored by changes in EGFP fluorescence using a plate reader (Fig. 2A). Increase of fluorescence with time demonstrates that the fusion proteins were produced - a conclusion that was further corroborated by fluorescence scanning of the SDS-PAGE gels loaded with translation mixture (Fig. 2B). In all cases full-length fusion proteins were produced yielding from 30 to 120 µg/ml. These yields are lower than reported with systems primed with plasmid based templates but still sufficiently high for biochemical and interaction analysis [4].

Finally we wanted to test whether expressed proteins could be isolated from the translation reaction mixtures. For that we incubated the reactions with single chain antibody to GFP cross-linked to the solid support (brand name GFP-Cap). Upon washing the proteins were eluted with SDS-PAGE loading buffer and resolved via SDS-PAGE gel. Figure 2C shows that with one exception all proteins could be isolated at detectable levels. Therefore we concluded that the developed procedure was sufficiently robust for parallelized expression and purification of the proteins.

### In vitro expression of L. tarentolae proteins

In the next step we investigated whether our methodology could be used for direct expression and biochemical analysis of *Leishmania* proteins using genomic DNA as a template. Due to our interest in regulators of translation initiation and elongation in *Leishmania*, we selected 31 genes coding for putative translational factors and phosphatases. As the *L. tarentolae* genome is not fully annotated, we used annotation of the *L. major* and *L. infantum* genomes to find corresponding homologs in the *L. tarentolae* genome.

**Figure 3:**
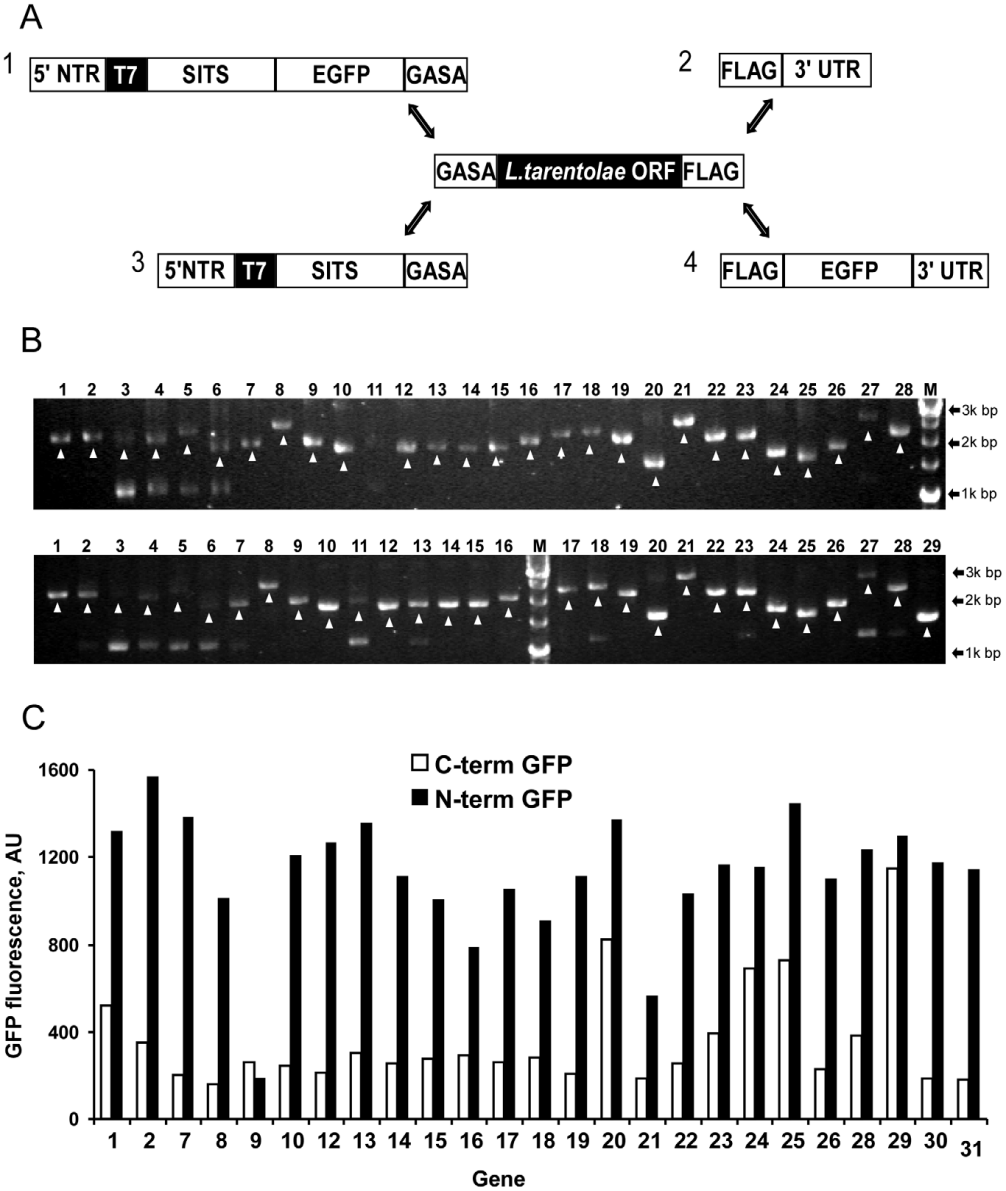
OE-PCR-synthesis and *in vitro* translation of *egfp* tagged *L. tarentolae* genes. (A) Structure of fragments used for OE-PCR assembly of *L. tarentolae* genes N- or C-terminally tagged with egfp. Full length templates with N-terminal EGFP were obtained using fragments 1 and 2 while the C-terminal fusion was obtained with fragments 3 and 4. Sequences of resultant assemblies are shown in Fig. S1. Abbreviations: 5 -NTR- 5′ not transcribed region, 3′ UTR - 3′ untranslated region, T7 – T7 promoter, GASA and FLAG are compatible sequences employed for OE-PCR and encoding GASAGSGS and DYKDDDDK peptides respectively (see Fig. S1B,C for details). (B) 0.7% agarose gel analysis of the OE-PCR products. The C-terminal EGFP templates are shown on the upper panel while the lower panel shows the N-terminally tagged constructs. White arrowheads denote specific products. (C) EGFP fluorescence of the reaction mixtures primed with templates shown in (B). Reactions primed with N- terminal EGFP fusions are displayed as filled bars while the fluorescence of C terminal fusion is shown as empty bars. All templates were normalized and added in equal molar concentration to the translation reaction.

The selected genes were amplified from genomic DNA and each product was extended with two different sequences adding EGFP tags on either the N- or C-terminus of the open reading frame (Fig. 3A). We chose to test both orientations of the tag to assess its influence on the protein folding, and to test the flexibility of the developed template synthesis protocol. OE-PCR resulted in the successful amplification of 60 fusion genes with comparable yields (Fig. 3B). The concentrations of PCR products were normalized before priming LTE lysate, and subsequent progress of the protein synthesis was monitored by fluorescence. Figure 3C compares fluorescent yields for each tag location. Placing the fluorescent domain at the end of the fusion polypeptide significantly reduced the total fluorescence yield. This is a known phenomenon related to the influence of the folding intermediates of the payload on the folding of EGFP, and was exploited to monitor stability of the recombinant proteins in *in vivo* selection systems [12]. Taking this into account, the initial screen suggests that the *L. tarentolae* homologues of eIF1a, LeisheIF4E1, eIF5a and TF SUI1 display rapid folding kinetics and high structural stability. This conclusion is also supported by an apparent inverse correlation between protein length and the fluorescence of the fused N-terminal EGFP relatively to the C-terminal construct. For instance, the shortest member of the screen TF SUI1 (gene 29) displayed a nearly equal fluorescence for the both tagged forms.

**Figure 4:**
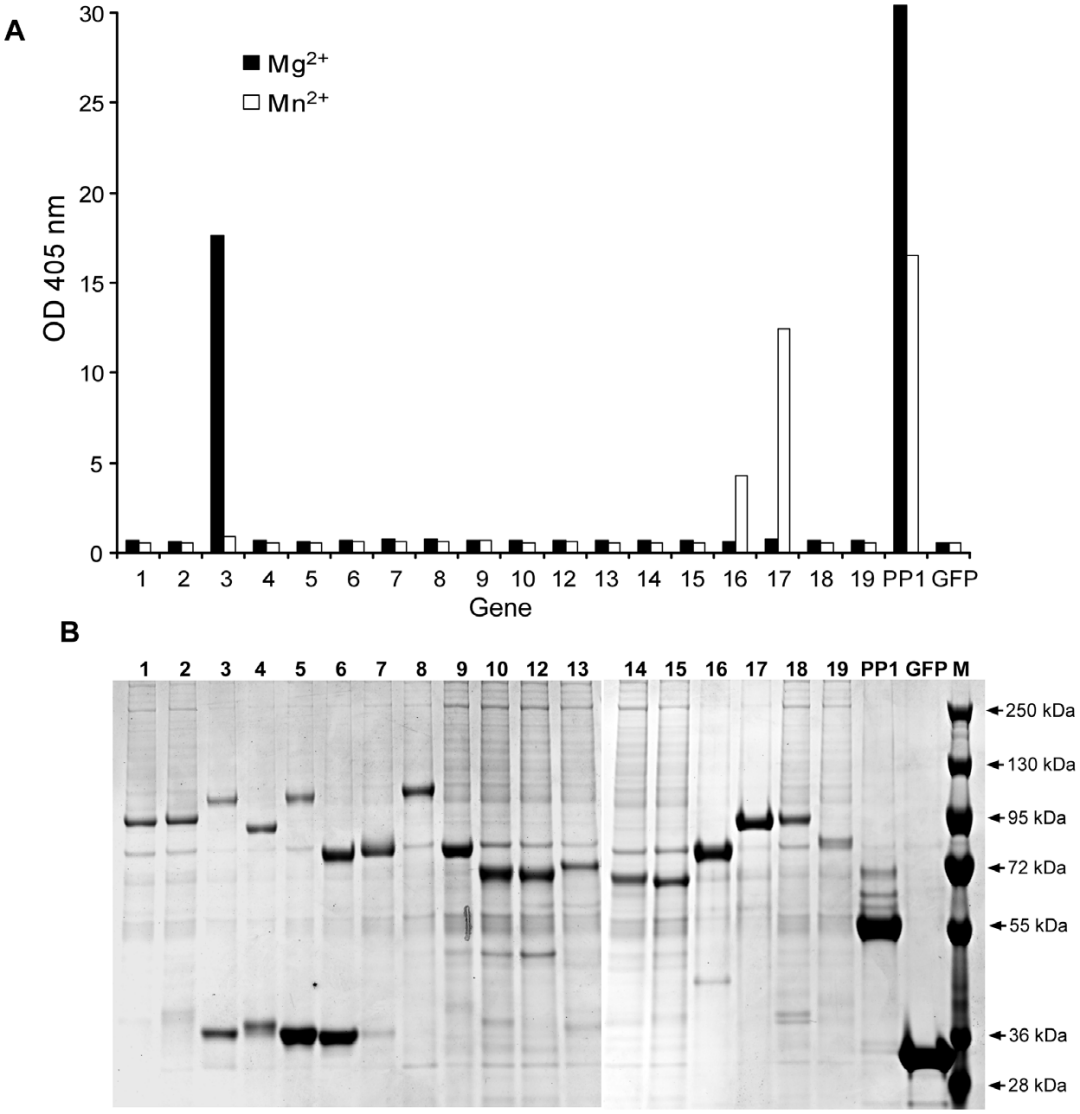
Activity and integrity of recombinant EGFP-tagged putative phosphatases. (A) Result of the pNPP hydrolysis assay performed with GFP-Cap matrix carrying EGFP-tagged putative phosphatases isolated from the *in vitro* translation reaction. The filled bars indicate assays performed in Mg^2+^-contaning buffer while the empty bars denote assay performed in Mn^2+^ - containing buffer. C-terminal EGFP-tagged catalytic subunit of human PP1 serine/threonine phosphatase and EGFP were included as positive and negative controls respectively. (B) Coomassie stained SDS-PAGE of EGFP-tagged phosphatases isolated from *in vitro* translation reaction on GFP-cap affinity resin and used for enzymatic analysis in (A). The 36kDa proteins in lanes 2–6 are likely to represent spurious translation products derived from amplification of the fragment 1 depicted in the figure 3A.

In the next step, we wanted to assess the biological activity of the *in vitro* expressed proteins. We chose to work only with the subset of proteins representing putative phosphatases C-terminally tagged with EGFP. For protein purification we again employed the GFP-Cap matrix system. Although this combination of C-terminal tag and purification procedure reduces the total isolatable amount of recombinant protein, it ensures the homogeneity of the sample due to the exclusion of potential premature termination products. Incubation of the translation lysates with GFP-Cap beads led to quantitative removal of the EGFP fluorescence from the lysate (data not shown). To assess the catalytic activity of the putative phosphatases we performed an *in vitro* phosphatase activity assay which utilizes the non-specific phosphatase substrate p-Nitrophenyl Phosphate (pNPP) [13]. As positive and negative controls we chose the well characterized phosphatase PP1 and EGFP respectively, which were expressed in LTE and purified on the GFP-Cap beads as per the rest of the experimental enzymes [14]. As many phosphatases require Mg^2+^ or Mn^2+^ for activity we performed the assay in two different buffers containing one or the other of these ions. An initial assay in the Mg^2+^ containing buffer revealed a high activity of phosphatase #3 (PTP-1 like protein, putative protein tyrosine phosphatase). Repeating the experiment in buffer containing Mn^2+^ revealed two more highly active phosphatases #16 and #17 (putative serine-threonine protein phosphatases PP2C and PP5 respectively), while the activity of PTP-1 was lost (Fig. 4A). The fact that initial analysis confirmed activity in only a subset of the putative phosphatases may indicate that we either did not find the correct buffer composition, or the putative assignment of the tested genes as phosphatases is incorrect. A less likely scenario is that the EGFP fusion and the peptide derived from the translational leader affected activity of the recombinant fusion proteins. These results indicate that the methodology developed in the current study is sufficiently versatile to express a large number of ORFs starting from genomic DNA. This is expected to accelerate *Leishmania* proteome deorphanisation by providing a rapid access to recombinant *Leishmania* proteins.

Next we wanted to visualize the expressed putative phosphatases and assess the amounts and homogeneity of the recombinant proteins. To this end we eluted the recombinant proteins from the GFP-Cap matrix and resolved them on SDS-PAGE gel subsequently stained with Coomassie. As can be seen in figure 4B, in all cases recombinant fusion protein could be readily detected, however the amounts varied significantly (0.4 to 6 µg as estimated by densitometry of the gel bands). This indicates that the cell-free expression and purification protocol presented in this study delivers sufficient amounts of *Leishmania* protein for biochemical analysis.

### Comparative analysis of protein folding activity in Leishmania and E. coli cell-free systems

**Figure 5:**
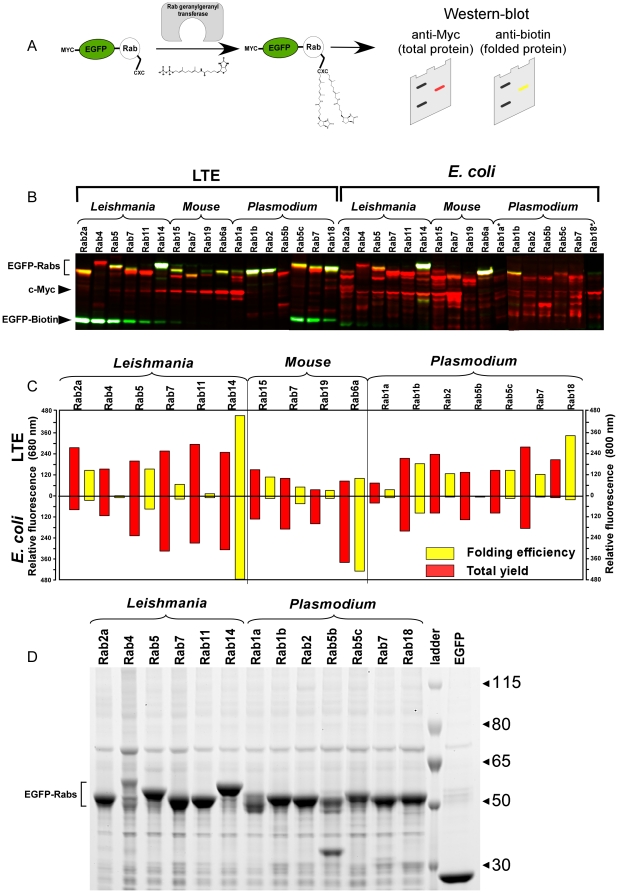
Expression of RabGTPases of different origins in LTE and *E. coli* cell-free systems and analysis of their folding state. (A) Schematic representation of Rab *in vitro* prenylation assay used to monitor folding state of GTPases expressed in different cell-free systems. Detection of myc-tag and biotin-geranyl-conjugated proteins was achieved by Western blotting with mouse anti-myc primary/IR680nm-labelled anti-mouse secondary antibodies and IR800nm-labelled streptavidin. The resulting blots were scanned using the Odyssey Imaging System (Li-Cor). (B) Western blot analysis of *in vitro* translation reactions primed with PCR products coding for myc-EGFP-Rab fusions of different origins. The gels were loaded with 0.4 µl of LTE and 2.13 µl of *E. coli* translation reaction mixtures. The membranes were blotted with anti-myc primary/IR680nm-labelled anti-mouse secondary antibodies (red) and IR800 nm-labelled streptavidin (yellow). The gels were also co-loaded with the decreasing concentrations of biotinylated EGFP and myc-tagged 34 kDa protein as transfer and loading standards (indicated by arrowheads on the left hand side). The picture represents overlay of images scanned in two wavelengths. Asterisks indicate translation of templates with shorter 3′ UTR. Shorter UTRs may influence the expression levels due to reduced mRNA stability. (C) Quantification of the expression and folding efficiencies of RabGTPase expressed in *E. coli* and LTE cell-free systems. The bars represent the integrated intensities of IR680 and IR800 dyes reflecting biotin and myc signals respectively. The background signals of respective channels were assigned zero intensity. (D) Coomassie stained SDS-PAGE gel loaded with EGFP-Rab GTPases eluted from GFP-Cap matrix. For Rab GTPase expression 200µl LTE *in vitro* translation reactions primed with PCR products coding for *L. tarentolae* and *P. falciparum* RabGTPases were isolated on GFP-cap matrix and analysed by SDS-PAGE as described in the Materials and Methods section. The expected migration position of EGFP-Rab fusion proteins is indicated on the left side of the gel.

The data presented so far demonstrates that the LTE system can translate genes of different origins into proteins and is sufficiently versatile to be used for high throughput protein expression. The simplicity of lysate preparation and the high expression level place this system ahead of most of eukaryotic cell-free expression systems [4]. The only system that can rival LTE in terms of scalability, yield and production cost is the *E. coli* cell-free system [15]. Next we decided to directly compare the ability of LTE and *E. coli* cell-free systems to produce natively folded proteins of different origins. To this end we focused on the family of RabGTPases that represent the key regulators of vesicular transport in all known eukaryotic organisms [16]. The structurally and functionally conserved RabGTPases underwent expansion during evolution with 11 proteins identified in *S. cerevisiae* and more than 60 proteins in humans [17]. Despite their functional conservation RabGTPases can display as little as 30% sequence identity among each other within a particular species [17]. For our comparative analysis we amplified the genes coding for mammalian, *L. tarentolae* and *P. falciparum* RabGTPases as described in the materials and methods section. All genes were fused to a 5′ SITS-myc-tag-egfp assembly using overlap-extension PCR as described above, and the PCR products were used to prime transcriptionally-translationally linked LTE and *E. coli* cell-free systems respectively. In order to analyze the folding efficiency of individual RabGTPases we took advantage of the protein prenylation assay developed previously in our group [4], [18]. The assay takes advantage of the fact that RabGGTase recognizes the three-dimensional structure of RabGTPases and therefore discriminates between folded and unfolded GTPases [19]. Further, since the interface between RabGTPases and RabGGTase is conserved in eukaryotic evolution, the mammalian enzyme can efficiently process RabGTPases derived from the unicellular organisms [20]. In this assay covalent attachment of biotin functionalized isoprenoid to RabGTPases by Rab geranylgeranyl transferase (RabGGTase) provides a measurement of protein folding (Fig. 5A). Presence of myc epitope on the N-terminus of the fusion proteins was used to estimate the absolute expression yields and formation of the full length polypeptide via Western blotting, additionally blotting with fluorescent streptavidin conjugates allowed discrimination between folded and unfolded products (Fig. 5B). This experiment demonstrates that when primed by PCR products in a form of a crude reaction mixture, the total yield of *Leishmania* and mammalian GTPases was on average 5 fold higher in LTE as compared to the *E. coli* cell-free system. Additionally, the GTPases of *P. falciparum* displayed a large number of premature termination products when expressed in the *E. coli* cell-free system (Fig. 5B,C). Analysis of the folding efficiencies using the *in vitro* prenylation assay demonstrated that the LTE system produced a majority of GTPases in the natively folded state, while *E. coli* was far less efficient in folding these proteins (Fig. 5C). Interestingly Rab4 of *L. tarentolae* produced in either of the systems could not be prenylated *in vitro*. This may indicate that the initial assignment of this ORF as RabGTPase may be incorrect or that its folding *in vivo* is facilitated by factors not present in the *Leishmania* cell-free system.

To assess the integrity of synthesized polypeptides we purified *L. tarentolae* and *P. falciparum* GTPases expressed in LTE and analyzed them via SDS-PAGE analysis. The EGFP tagged RabGTPases were isolated on the GFP-cap beads and subjected to the SDS-PAGE analysis as described above. As can be seen in figure 5D the majority of RabGTPases migrated at their expected molecular weights with only *L. tarentolae* Rab4 appearing as several bands and Rab5B of *P. falciparum* displaying an additional band of ca. 38kDa. Remarkably GTPases of *P. falciparum* were expressed in LTE as efficiently as the homologous RabGTPases.

### Translation efficiency of SITS containing templates in Leishmania and E. coli cell-free systems

**Figure 6:**
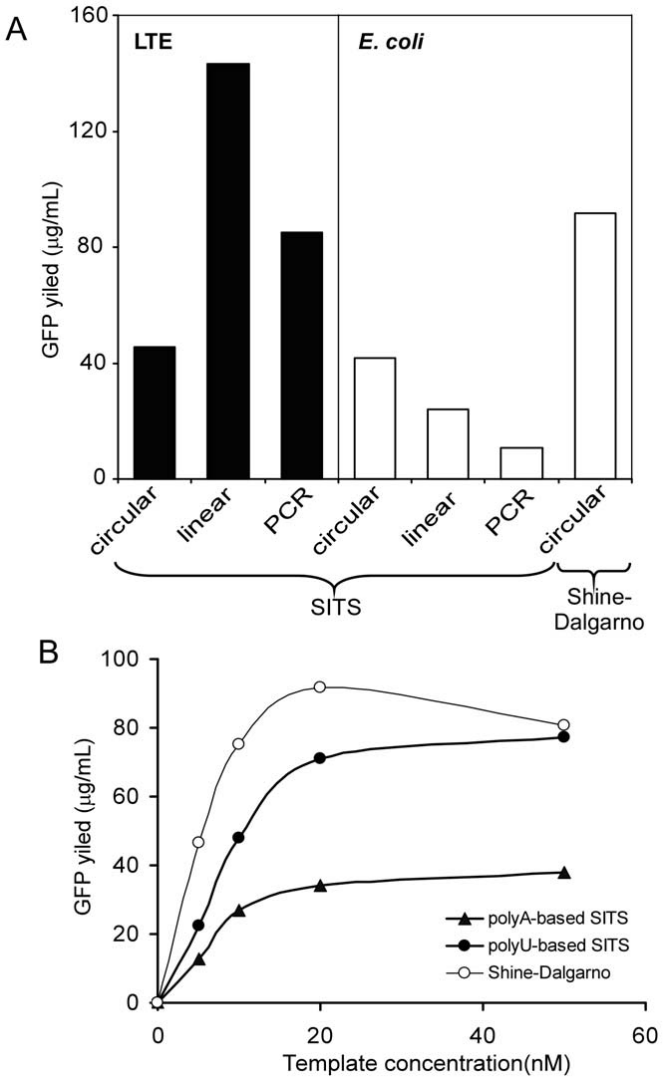
Translation efficiency of SITS containing templates in *Leishmania* and *E. coli* cell-free systems. (A) Comparison of batch cell-free translation reactions primed with different EGFP-encoding templates. For comparison 20 µl of translation reaction mixtures were primed with 20 nM of plasmid DNA or 10% (v/v) of crude PCR reaction mixture. Translation reactions were carried out at 26°C or 30°C for *Leishmania* and *E. coli* systems respectively until no additional GFP fluorescence was generated. GFP concentration in the endpoint reactions was estimated by 488nm excitation/509nm emission spectrum (B) As in A but reactions were primed with different amounts of circular plasmids with different translation initiating sequences.

In the experiments described above we used the SITS leader to drive the translation of PCR-generated linear templates in LTE and *E. coli* lysates. Although these experiments were focused on the analysis of folding efficiencies, total expression yield provides an additional important measure of system performance. Hence, we decided to compare the classic Shine-Dalgarno ribosome binding site with the poly(A)-based SITS leader used in this study. To this end we measured the EGFP expression yield in the *E. coli* cell-free system primed with circular plasmids coding for EGFP and carrying poly(A)-based SITS or Shine-Dalgarno sequences. For an additional comparison of template performance we primed *Leishmania* and *E. coli* systems with circular and linear plasmids coding for SITS-egfp, and also with unpurified PCR-amplified SITS-ORF assembly obtained from the same plasmid. The results (depicted in the figure 6A) demonstrate that the *Leishmania* system performed best when primed with linearised plasmid. The PCR products were translated with 60% efficiency of linearised plasmid, and the circular plasmid displayed only 40%. In contrast the *E. coli* system performed best when primed with circular template and the PCR product was only 25% as efficient. The likely explanation is degradation of linear DNA and mRNA molecules by elevated (exo)nuclease activity present in *E. coli* lysate [21]. The Shine-Dalgarno containing sequences performed approximately twice better in *E. coli* than poly(A) SITS sequences. However, we wanted to ascertain that this was not due to the different concentration dependence of the leaders. To analyze this we performed the *in vitro* translation reactions in *E. coli* extract with different concentrations of circular templates. Figure 6B shows that all sequences showed similar concentration dependence and poly(A) SITS was at least 50% less efficient than the Shine-Dalgarno sequence. However the SITS carrying unstructured (UUUUA)_11_ [4] sequence was almost as efficient as Shine-Dalgarno sequence (Fig. 6B). Although the expression yields achieved in our *E. coli* cell-free system are lower than reported elsewhere, the relative expression efficiencies of SITS leaders are likely to be the same across the systems of different manufacture.

## Discussion

In the current study we used recently developed Species Independent Translational Leaders (SITS) to initiate high efficiency translation in lysates prepared from *Leishmania tarentolae* and *E. coli* cells. These leaders demonstrated efficient translational initiation largely independent of the following open reading frame, thus enabling production of biochemically relevant amounts of recombinant protein in both LTE and in *E. coli* cell-free systems. The implications of these experiments are several fold. Firstly, our results demonstrate that using SITS it is possible to prime cell-free systems of different origins with the same template. This was not previously possible due to the highly species-dependent nature of the available translation initiation sequences. The availability of SITS will facilitate the analysis of translation mechanisms in different cell-free system and direct comparison of cell-free systems with each other.

We demonstrated that the LTE protein expression system can be used for direct genome to proteome conversion in *Leishmania*. In the arbitrary selected set of 31 genes, 30 were successfully amplified and expressed in LTE, and the activity of 19 proteins was assayed. This shows that the developed strategy is simple and versatile, and can potentially be used to obtain complete expressed proteomes of parasitic organisms. Based on the obtained experimental data we projected that expression of a kinetoplastida proteome containing ca. 6000 genes could be done by a single person in 14 days with no laboratory automation. The main component to the price is the cost of the comparatively short oligonuclotides (average of 43bp) used for overlap extension PCR. This cost is expected to decrease rapidly in the future. Even more importantly the analysis of small GTPases produced in LTE and *E. coli* demonstrated that the LTE consistently produced better folded proteins.

In summary we conclude that the developed approach represents a major technological advancement in the proteome analysis of parasitic organisms that will accelerate elucidation of biochemistry of parasitic organisms, identification of new drug targets and development of vaccines.

## Materials and Methods

### Preparation of the supplemented Leishmania tarentolae Extract (LTE)

*Leishmania* cell culture propagation and cell lysate preparation was performed as described in [4]. The obtained rebuffered lysate was supplemented to 40% (v/v) of its volume with feeding solution containing 8.5 mM ATP, 3.17 mM GTP, 1.25 mM spermidine, 10 mM DTT, 200 mM creatine phosphate, 12.5 mM Mg(OAc)_2_, 32 mM HepesKOH pH 7.6, 5% (v/v) PEG 3000, 5.25× protease inhibitor (Complete™ EDTA-free, Roche), 0.68 mM of each amino acid, 2.5 mM of each UTP and CTP, 0.05 mM anti-splice leader DNA oligonucleotide, 0.5 mg/ml of T7 RNA polymerase, 200 U/ml of creatine phosphokinase. The supplemented lysate was aliquoted, snap-frozen in liquid nitrogen and stored at −80°C.

### Preparation of PCR products for OE-PCR

In all cases PCR reaction mixture contained 0.2 mM of each of the dNTPs (Fermentas) and 2 U/100 µl of Phusion Polymerase (NEB) in 1× HF Phusion reaction buffer (NEB). As a PCR template, plasmid 1751 (Fig. S1A) encoding for T7promoter-SITS-gfp (for universal 3′ fragments) or *L. tarentolae* genomic DNA (for variable fragments) at concentration of 1 ng/µl were used. All primers used in this work are listed in the Supplementary information section (tables S1, S2 and S3) Universal fragments were synthesized at 500 nM concentration of the primer pairs 511 and 8928, 511 and 8927, 8929 and 2511 or 8926 and 2511 for the fragments 1 to 4 respectively (Fig. 3A). PCR thermal cycling consisted of initial DNA denaturation at 98°C for 2 minutes, 30 cycles of denaturation at 98°C for 10 seconds, annealing at 60°C for 30 seconds and elongation at 72°C followed by final elongation at 72°C for 2 minutes.

The genomic PCR was performed similarly to the plasmid-programmed reaction with the modification in the annealing step (temperature decrement of 1° from 68°C to 60°C on the first eight cycles was used, then 27 cycles at 60°C, annealing for 50 seconds) and prolonged to 40 seconds elongation step. In addition, the fragment encoding for PP1 phosphatase catalytic subunit (GeneBank ID NP_002699) was amplified from PP1-encoding plasmid with primers 9176 and 9177 using PCR conditions as given for genomic PCR. Primers 8817–8878 at 300 nM concentration were used in amplification of the genes 1–31 with 35 cycles. Synthesized universal fragments were gel-purified (QIAquick Gel Extraction Kit, Qiagen). Variable ORF-encoded fragments were freed from residual primers by treatment with 15 U/ml Exonuclease I (NEB), which was added directly to the final PCR reaction mixture, for 30 minutes at 37°C followed by nuclease inactivation at 85°C for 30 minutes.

### Synthesis of translational templates by overlap extension (OE-PCR) PCR

To obtain the libraries of DNA templates two universal fragments (1, 2 or 3, 4, Fig. 3A) were combined with one of the 31 variable PCR fragments using OE-PCR. As a result two sets of 31 templates encoding for N- and C-terminal EGFP fusions were obtained (Fig. S1B;C). OE-PCR reaction mixture contained 0.2 mM of each of dNTP, 2.5 U/100 µl of Taq polymerase (NEB), 500 nM of 511 and 2511 primers, 5 nM of gel-purified universal fragments and 2.5% (v/v) of nuclease treated PCR reaction mixture containing one of the 31 variable fragments. Thermal cycling included initial denaturation at 95°C followed by 30 cycles of denaturation at 94°C for 30 seconds, annealing at 50°C for 30 seconds and elongation at 72°C for 3 minutes followed by final elongation at 72°C for 5 minutes. To assess the efficiency and specificity of the PCR reaction 2 µl of the resultant OE-PCR mixture was analyzed on 0.7% agarose gel (Fig 3B).

### Cell-free translation in LTE

Crude OE-PCR mixtures were used to prime the translation reaction. The translation reaction mixture contained 70% (v/v) of the supplemented LTE, 10% (v/v) of OE-PCR mixture or 10 nM of the linearized plasmid as a template and was adjusted to the final volume with nuclease-free mQ water (Ambion). For the comparative analysis of expression efficiencies of *L. tarentolae* proteins the concentrations of PCR products were standardised. The PCR products 3–6 were excluded from the analytical translation set due to the presence of low molecular weight PCR by-products representing the SITS-EGFP assembly. The protein synthesis was carried out at 26°C in a thermo cycler (MyCycler, BioRad) for 2 hours. To control the progress of the synthesis reaction, a 20 µl aliquot was incubated in a well of a 384-well plate at 26°C and EGFP fluorescence was monitored using a fluorescence plate reader (Synergy 4, BioTek).

### Analysis of in vitro translated putative phosphatases

*In vitro* translation mixtures were adjusted with NaCl and Triton ×100 to 150 mM and 0.1% (v/v) concentration respectively. GFP-Cap resin (50% slurry, Jena Bioscience) was mixed with equal volume of 30% glycerol to prevent sedimentation of the matrix during pipetting. 30 µl of the resulted slurry was introduced to 200 µl of the translation reaction mixture and incubated at room temperature for 25 minutes with active shaking. Resin was pelleted at 2000 g for 2 minutes, the supernatant was removed and its fluorescence was measured in the plate reader. The resin pellets were washed 3 times with 1 ml of washing buffer (20 mM HepesKOH pH 7.5, 500 mM NaCl, 2 mM MgCl_2_, 2 mM DTT) and once with the washing buffer containing 150 mM NaCl. The phosphatase assay was performed directly with the proteins immobilized on the GFP-Cap beads. For this, the washed beads were resuspended in 50 µl of 50 mM of pNPP (Sigma) in reaction buffer and incubated for 30 minutes at 30°C with agitation. Subsequently, the beads were pelleted by centrifugation as above and an aliquot of the reaction mixture taken for spectrophotometric analysis. The beads were washed as described above to remove the hydrolysis product of the pNPP and MgCl_2_ was replaced by an equal concentration of MnCl_2_. The phosphatases activity of the washed beads in the presence of Mn^2+^ was assayed as described above. The collected reaction mixtures were diluted if needed, placed in transparent 96-well plate (Nunc) and their absorption was measured at 405 nm using plate reader (Synergy4, BioTek).

Finally, the bound material was eluted from the beads using 80°C 1× SDS loading buffer (Invitrogen) and resolved on 4–12% PAGE (NuPage, Invitrogen), followed by protein band detection with Coomassie Blue R (Sigma) staining.

### Cell-free translation in E. coli extract

Translational active S30 lysate of *E. coli* was prepared according to [22]. After optimization of K^+^ and Mg^2+^ concentrations, the *E. coli* cell-free translation yielded 24 µg/ml of fluorescent active EGFP when programmed with 20 nM of linear SITS-prefaced EGFP-coding DNA template. Optimized *E. coli in vitro* translation reaction mixture contained 35% (v/v) of S30 lysate, 45% (v/v) of feeding solution (4.9% PEG8000, 368 mM KOAc, 17.4 mM Mg(OAc)_2_, 218 mM Hepes-KOH pH 8.0, ×5.9 protease inhibitor, 0.24 mg/ml folinic acid, 4.9 mM DTT, 2.93 mM ATP, 1.35 mM of each of CTP, GTP and UTP, 4.9 mM phosphoenol pyruvate, 4.9 mM acetyl phosphate, 3.67 mM of each of arginine, cysteine, tryptophan, methionine, aspartic acid, glutamate and 1.22 mM of the rest of canonic amino acids), 0.08 mg/ml of T7 RNA polymerase, 0.4 U/µl of RNAse inhibitor (RNase OUT, Invitrogen), 0.5 mg/ml of total *E. coli* tRNA (Sigma), 0.04 mg/ml Pyruvate Kinase and 10% (v/v) of crude PCR mixture. Translation reactions were performed in 384-well plate in the fluorescence plate reader at 30°C.

### Synthesis of RabGTPases templates

A set of templates encoding N-terminal EGFP fusions with six putative Rabs from *L. tarentolae*, four mammalian Rabs or seven *P. falciparum* Rabs (see tables S3 and S4 for details) were synthesized by OE-PCR as described above. To prepare ORF-coding fragments for OE-PCR, *Leishmania* Rab sequences were amplified from genomic DNA using primers 1–12 (Table S3). Plasmids encoding EGFP-Rab fusions were used as templates to synthesize ORFs of mammalian Rabs with primers 3510 and 8581 (Table S2). To obtain PCR products encoding *Plasmodium* Rabs, total mRNA of *P. falciparum* was reversely transcribed into cDNA using oligo dT primer and the resultant cDNA library was used as a template for amplification of individual Rabs with primers 13–26 (Table S3). The universal fragments used for template preparation were obtained by PCR on plasmid 1751 (Fig. S1A) with primers 511/2522, 511/9152 and 33/2511 for fragments from 5 to 7 respectively (Fig. S2). To assemble the full-length templates, the PCR products encoding *Leishmania* Rabs were fused by PCR with the universal fragment 5 (Fig. S2A), while the fragments containing mammalian Rab were fused with fragment 6 (Fig. S2D). The fragments encoding *P. falciparum* Rab were fused to the fragment 5 (Fig S2B). Since these templates have short 5′ UTR that may reduce the stability of the mRNAs when translated in *E. coli* lysate, we extended 3′ UTRs by 200 bp for five of the templates using universal fragment 7 (Fig. S2C). Sequence of Rab-containing PCR assemblies is shown in Fig. S3.

### In vitro prenylation assay and protein folding analysis

The Rab prenylation reaction was performed as in [4] with minor modifications. Upon completion of protein synthesis, 2 µl (LTE) or 4 µl (*E. coli*) aliquots of translational reaction as well as corresponding volume of positive and negative control reactions were mixed with the master mix to obtain 15 µl of prenylation reaction. As a negative control we used lysates with no template added, whereas positive control contained 1 pmol of purified recombinant canine Rab6A spiked into the respective cell-free lysate. Final concentration of components in the prenylation reaction were as follows: 20 µM GDP, 2 mM DTT, 2 µM of each of recombinant proteins RabGGTase and REP1 (Jena Bioscience), 5.5 µM of biotin geranylgeranyl pyrophosphate (Jena Bioscience), 2 mM of MgCl_2_, 40 mM NaCl buffered with 25 mM of HepesKOH pH 7.6. The prenylation was allowed to progress for 4 hours at 22°C, then 3 µl (LTE) or 8 µl (*E. coli*) of it was resolved on 4–12% PAGE (NuPage, Invitrogen). After electrophoresis, proteins were transferred to nitrocellulose membrane (Pall) by semidry transfer method. The membrane was blocked and incubated with 1∶1000 dilution of mouse anti-myc antibodies (Cell Signalling Technology). Then the membrane was transferred to the mixture of 1∶10,000 dilution of anti-mouse IR680nm-labelled antibodies and IR800nm-labelled streptavidin (Li-Cor). The membrane was washed, dried and scanned on Odyssey Imaging System (Li-Cor). The data were processed with Odyssey Application Software V3.0 (Li-Cor). *E. coli* controls displayed a single streptavidin-reactive 15 kDa band representing an *E. coli* biotinylated protein. Positive controls confirmed that components of translation reactions do not interfere with prenylation (data not shown).

## Supporting Information

Figure S1 Sequence of DNA templates used for priming cell-free translation reactions
(0.08 MB DOC)

Figure S2 Structure of fragments used for OE-PCR-based assembly of transcription templates coding for N-terminal GFP fusions with Rab GTPase genes of different organisms
(0.04 MB DOC)

Figure S3 Sequence of Rab-encoding DNA templates used for priming cell-free translation reactions.
(0.12 MB DOC)

Table S1 (0.11 MB DOC)

Table S2 (0.04 MB DOC)

Table S3 (0.05 MB DOC)

Table S4 (0.03 MB DOC)
