# Supplementary material for: Towards the Construction of Expressed Proteomes Using a Leishmania tarentolae Based Cell-Free Expression System
Source: PLoS One. 2010 Dec 21;5(12):e14388. doi: 10.1371/journal.pone.0014388 (PMC3006200; doi:10.1371/journal.pone.0014388)
Supplement: Figure S1 — Sequence of DNA templates used for priming cell-free translation reactions (0.08 MB DOC) [file pone.0014388.s001.doc]

| 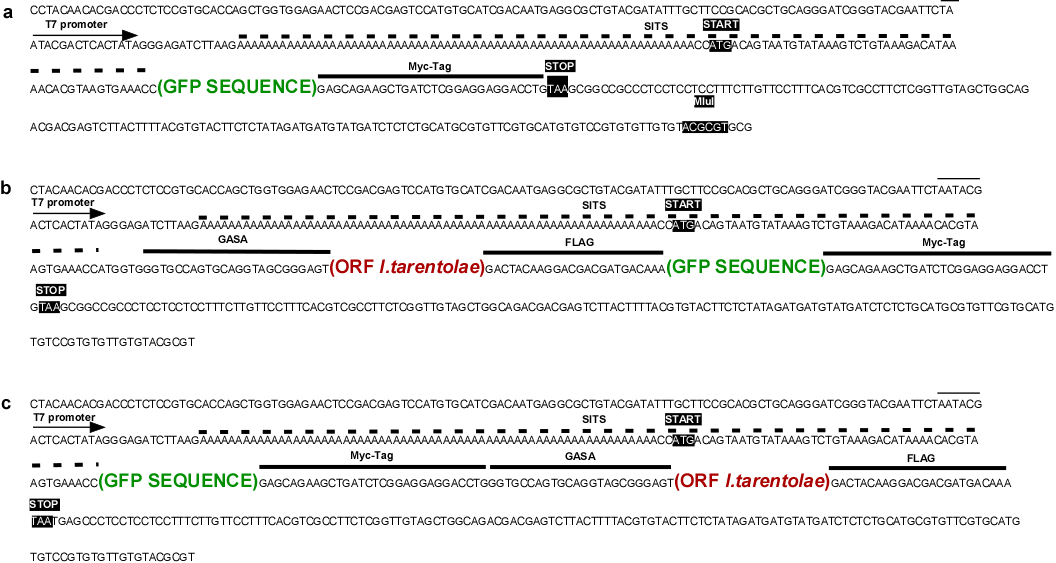 |
| --- |
| ***Figure S1****.* ***Sequence of DNA templates used for priming cell-free translation reactions. (a)*** *Section of**sequence of plasmid 1751 that was used as a template for construction of transcription templates.* ***(b)*** *The PCR template encoding for C-terminal ORF-GFP fusion* ***(c)*** *as in b but encoding N-terminal GFP tag. T7 promoter, SITS (dashed line), myc-tag encoding sequence, FLAG and GASA bridging sequences are indicated.* |
