# Supplementary material for: Towards the Construction of Expressed Proteomes Using a Leishmania tarentolae Based Cell-Free Expression System
Source: PLoS One. 2010 Dec 21;5(12):e14388. doi: 10.1371/journal.pone.0014388 (PMC3006200; doi:10.1371/journal.pone.0014388)
Supplement: Figure S2 — Structure of fragments used for OE-PCR-based assembly of transcription templates coding for N-terminal GFP fusions with Rab GTPase genes of different organisms (0.04 MB DOC) [file pone.0014388.s002.doc]

| *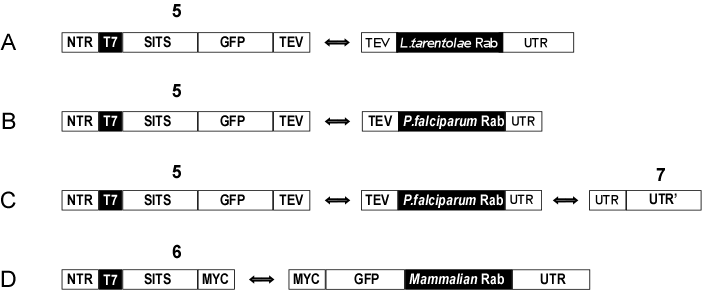* |
| --- |
| ***Figure S2****.* ***Structure of fragments used for OE-PCR-based assembly of transcription templates coding for N-terminal GFP fusions with Rab GTPase genes of different organisms****. (A) Assembling of the templates containing* Leishmania*, (B and C) as in A but for* Plasmodium *Rab GTPases and (D) mammalian Rab sequences. Abbreviations: 5’-NTR- 5’ not transcribed region, 3’ UTR -3’ untranslated region, T7 – T7 promoter, TEV- TEV protease cleavage site, MYC- myc tag are sequences employed for OE-PCR and encoding TEV protease cleavage site, c-Myc epitope (see Fig. S3 for details). Fragments are numbered as in Materials and Methods section.* |
