# Supplementary material for: Towards the Construction of Expressed Proteomes Using a Leishmania tarentolae Based Cell-Free Expression System
Source: PLoS One. 2010 Dec 21;5(12):e14388. doi: 10.1371/journal.pone.0014388 (PMC3006200; doi:10.1371/journal.pone.0014388)
Supplement: Figure S3 — Sequence of Rab-encoding DNA templates used for priming cell-free translation reactions. (0.12 MB DOC) [file pone.0014388.s003.doc]

| *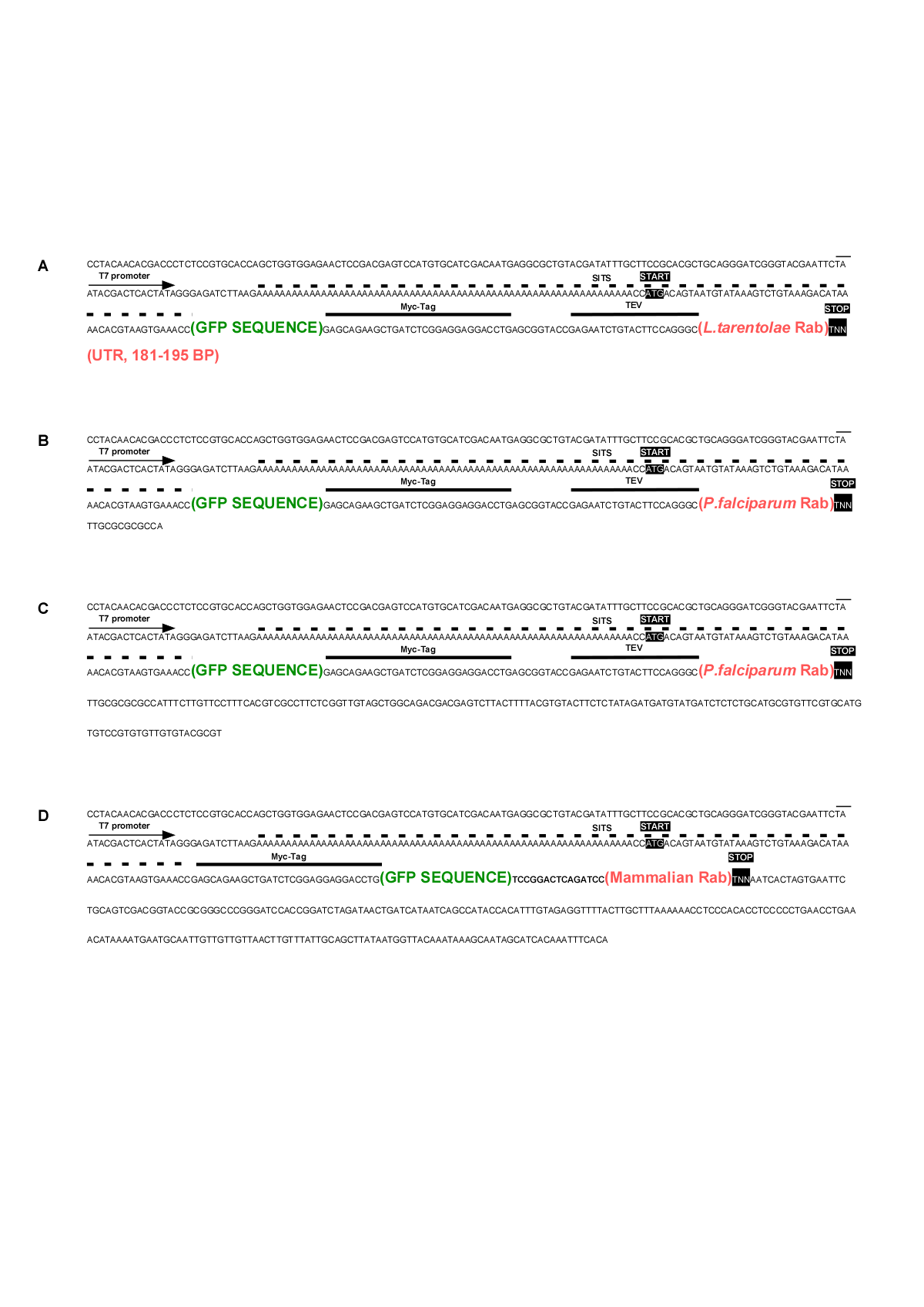* |
| --- |
| ***Figure S3****.* ***Sequence of Rab-encoding DNA templates used for priming cell-free translation reactions.*** *Sequences of**PCR templates encoding N-terminal GFP fusions with (A)* Leishmania *(B, C)* Plasmodium *and(D) mammalian Rab GTPases are shown. TEV indicates sequence coding for TEV-protease cleavage site. The rest of annotations are as in Fig S1.* |
