# Supplementary material for: Towards the Construction of Expressed Proteomes Using a Leishmania tarentolae Based Cell-Free Expression System
Source: PLoS One. 2010 Dec 21;5(12):e14388. doi: 10.1371/journal.pone.0014388 (PMC3006200; doi:10.1371/journal.pone.0014388)
Supplement: Table S1 — (0.11 MB DOC) [file pone.0014388.s004.doc]

| **Primer**  **number** | **Gene ID** | **Sequence** | **gene number** |
| --- | --- | --- | --- |
| **8817** | **LmjF36.5370** | **GCCAGTGCAGGTAGCGGGAGT**ATGTGCGAAAACCAATTCAAG | **1** |
| **8818** | **LmjF36.5370** | **TTGTCATCGTCGTCCTTGTAGTC**CACCAACGAAGGCGAGAAG | **1** |
| **8819** | **LmjF32.0640** | **GCCAGTGCAGGTAGCGGGAGT**ATGTGCGAAAACCAATTCAAGG | **2** |
| **8820** | **LmjF32.0640** | **TTGTCATCGTCGTCCTTGTAGTC**CACCAACGAAGGCGAGAA | **2** |
| **8821** | **LmjF36.2180** | **GCCAGTGCAGGTAGCGGGAGT**ATGCGCCATGACGTCCCG | **3** |
| **8822** | **LmjF36.2180** | **TTGTCATCGTCGTCCTTGTAGTC**GCGCTTTGGTGCGCGGGC | **3** |
| **8823** | **LmjF08.0100** | **GCCAGTGCAGGTAGCGGGAGT**ATGGGTGGCGGTGCTTCC | **4** |
| **8824** | **LmjF08.0100** | **TTGTCATCGTCGTCCTTGTAGTC**CACAGGCAGCGTCCCCTC | **4** |
| **8825** | **LmjF13.0770** | **GCCAGTGCAGGTAGCGGGAGT**ATGTTTTACCGAGACACGCTGT | **5** |
| **8826** | **LmjF13.0770** | **TTGTCATCGTCGTCCTTGTAGTC**CATGACTTGCGTCGGGCT | **5** |
| **8827** | **LmjF27.2210** | **GCCAGTGCAGGTAGCGGGAGT**ATGCTGTCGAAGGTGAGC | **6** |
| **8828** | **LmjF27.2210** | **TTGTCATCGTCGTCCTTGTAGTC**CGGCCTCCTCAGCACA | **6** |
| **8829** | **LmjF35.4650** | **GCCAGTGCAGGTAGCGGGAGT**ATGGATACAAGGTGCTGCAC | **7** |
| **8830** | **LmjF35.4650** | **TTGTCATCGTCGTCCTTGTAGTC**CAGCAACCCTAGCCTTGC | **7** |
| **8831** | **LmjF9.055** | **GCCAGTGCAGGTAGCGGGAGT**ATGAAGCCCACCAATGCC | **8** |
| **8832** | **LmjF9.055** | **TTGTCATCGTCGTCCTTGTAGTC**CACATTTTCGTGTTTCTCTTTTC | **8** |
| **8833** | **LmjF15.0220** | **GCCAGTGCAGGTAGCGGGAGT**ATGGCAAGCACGAAAAGAGG | **9** |
| **8834** | **LmjF15.0220** | **TTGTCATCGTCGTCCTTGTAGTC**GTCGTGGCTCAAAGGATTTG | **9** |
| **8835** | **LmjF28.0690** | **GCCAGTGCAGGTAGCGGGAGT**ATGAGCGTCGACTCCATTATT | **10** |
| **8836** | **LmjF28.0690** | **TTGTCATCGTCGTCCTTGTAGTC**CGAGTAGAACTTCGGCTTCTTC | **10** |
| **8837** | **LmjF31.2630** | **GCCAGTGCAGGTAGCGGGAGT**ATGAGTGGACTTTCGAAGGTAG | **11** |
| **8838** | **LmjF31.2630** | **TTGTCATCGTCGTCCTTGTAGTC**ACACTCGAAGTCGTTCACCTC | **11** |
| **8839** | **LmjF34.0780** | **GCCAGTGCAGGTAGCGGGAGT**ATGAGCGAATCTGTGCTTCCT | **12** |
| **8840** | **LmjF34.0780** | **TTGTCATCGTCGTCCTTGTAGTC**CTTCCTCGCCGGGATAATC | **12** |
| **8841** | **LmjF25.1320** | **GCCAGTGCAGGTAGCGGGAGT**ATGACGACAACCGGCGG | **13** |
| **8842** | **LmjF25.1320** | **TTGTCATCGTCGTCCTTGTAGTC**TAGGAAATAGTCCGGTGTCTTCT | **13** |
| **8843** | **LmjF32.3040** | **GCCAGTGCAGGTAGCGGGAGT**ATGTCAACTGAAATATTGGACATT | **14** |
| **8844** | **LmjF32.3040** | **TTGTCATCGTCGTCCTTGTAGTC**CAGGAAGTAGGCTGGTACCTC | **14** |
| **8845** | **LmjF34.4190** | **GCCAGTGCAGGTAGCGGGAGT**ATGAACCTCGATGCGTGGG | **15** |
| **8846** | **LmjF34.4190** | **TTGTCATCGTCGTCCTTGTAGTC**TAGAAAATAGGCCGGCTGCT | **15** |
| **8847** | **LmjF30.0380** | **GCCAGTGCAGGTAGCGGGAGT**ATGCTACCGAAGCCGATCC | **16** |
| **8848** | **LmjF30.0380** | **TTGTCATCGTCGTCCTTGTAGT**CCGAGTTATTGCTCGCGTTAG | **16** |
| **8849** | **LmjF18.0150** | **GCCAGTGCAGGTAGCGGGAGT**ATGGAGGAGTCCGACCGC | **17** |
| **8850** | **LmjF18.0150** | **TTGTCATCGTCGTCCTTGTAGTC**AACTAGAAATGCACCGCATGC | **17** |
| **8851** | **LmjF26.2530** | **GCCAGTGCAGGTAGCGGGAGT**ATGCTCCTCACAGACCGTGG | **18** |
| **8852** | **LmjF26.2530** | **TTGTCATCGTCGTCCTTGTAGTC**CCTGCTACTGGTGTTAGATGACC | **18** |
| **8853** | **LmjF36.1980** | **GCCAGTGCAGGTAGCGGGAGT**ATGACTCAATCCAATATAAAGTGCT | **19** |
| **8854** | **LmjF36.1980** | **TTGTCATCGTCGTCCTTGTAGTC**AATGAGTGGTGTGACACCTTCC | **19** |
| **8855** | **LmjF16.0140** | **GCCAGTGCAGGTAGCGGGAGT**ATGCCGAAGAACATGGGTAAG | **20** |
| **8856** | **LmjF16.0140** | **TTGTCATCGTCGTCCTTGTAGTC**AAGGTTGTCGAGGGCAGCA | **20** |
| **8857** | **LmjF16.1600** | **GCCAGTGCAGGTAGCGGGAGT**ATGCAGTTTACCGTGGAGCAG | **21** |
| **8858** | **LmjF16.1600** | **TTGTCATCGTCGTCCTTGTAGTC**TTTGGGGAAGCGCCCCTT | **21** |
| **8859** | **LmjF1.0780** | **GCCAGTGCAGGTAGCGGGAGT**ATGGCGCAGAACGATAAGGT | **22** |
| **8860** | **LmjF1.0780** | **TTGTCATCGTCGTCCTTGTAGTC**CTCGCCAAGGTAGGCAGCA | **22** |
| **8861** | **LmjF28.153** | **GCCAGTGCAGGTAGCGGGAGT**ATGGAGACGGAGCAGGTGG | **23** |
| **8862** | **LmjF28.153** | **TTGTCATCGTCGTCCTTGTAGTC**CACTTGCTCACCAATGTTGG | **23** |
| **8863** | **LmjF27.1620** | **GCCAGTGCAGGTAGCGGGAGT**ATGTCAGCCCCATCGGC | **24** |
| **8864** | **LmjF27.1620** | **TTGTCATCGTCGTCCTTGTAGTC**AGACGCCTCGCCATGCTT | **24** |
| **8865** | **LmjF25.072** | **GCCAGTGCAGGTAGCGGGAGT**ATGTCGGACGAGGACCACG | **25** |
| **8866** | **LmjF25.072** | **TTGTCATCGTCGTCCTTGTAGTC**CTCCGCAGCATTCTTCGTTT | **25** |
| **8867** | **LmjF36.089** | **GCCAGTGCAGGTAGCGGGAGT**ATGACGCTCCGCACCCG | **26** |
| **8868** | **LmjF36.089** | **TTGTCATCGTCGTCCTTGTAGTC**CGCCAGCTCGTCCACCA | **26** |
| **8869** | **LinJ35_V3.400** | **GCCAGTGCAGGTAGCGGGAGT**ATGGCCTTCACTGGTCCGA | **27** |
| **8870** | **LinJ35_V3.400** | **TTGTCATCGTCGTCCTTGTAGTC**AACACTCATGTGGCGGTTCA | **27** |
| **8871** | **LmjF36.4540** | **GCCAGTGCAGGTAGCGGGAGT**ATGCGGAGGACCTCTGGG | **28** |
| **8872** | **LmjF36.4540** | **TTGTCATCGTCGTCCTTGTAGTC**CTCTGACAACAGCGCTGAGAGT | **28** |
| **8873** | **LinJ24_V3.1240** | **GCCAGTGCAGGTAGCGGGAGT**ATGGAGGATACCGTCGAGGC | **29** |
| **8874** | **LinJ24_V3.1240** | **TTGTCATCGTCGTCCTTGTAGTC**CAGAGAGTGAATCTCGAGATTGTT | **29** |
| **8875** | **LmjF03.0980** | **GGCCAGTGCAGGTAGCGGGAGT**ATGGCGTCTTACTGTGTTACAGA | **30** |
| **8876** | **LmjF03.0980** | **TTGTCATCGTCGTCCTTGTAGTC**GTCAGCATCCTCATCATCATCG | **30** |
| **8877** | **LmjF35.0370** | **GCCAGTGCAGGTAGCGGGAGT**ATGTCCGACTCCAACTGGAAG | **31** |
| **8878** | **LmjF35.0370** | **TTGTCATCGTCGTCCTTGTAGTC**TGCTGCGTACAACTCGGGG | **31** |

**Table S1**. **Primers used for amplification of L.tarentoale genes.** Even numbers denote forward primers while the odd refer to the reverse primers **.** 5’-overhang sequences used as complementary linkers for OE PCR are shown in bold. Gene ID column represents homologous genes from *Leishmania major (LmjF)* and *Leishmania infantum (LinJ)* genomes used for identification of *L.tarentolae* using sequencing data deposited on the [http://tritrypdb.org](http://tritrypdb.org/) server. Genes are numbered according to Fig 4 and 5 of the main text.
