# Supplementary material for: Towards the Construction of Expressed Proteomes Using a Leishmania tarentolae Based Cell-Free Expression System
Source: PLoS One. 2010 Dec 21;5(12):e14388. doi: 10.1371/journal.pone.0014388 (PMC3006200; doi:10.1371/journal.pone.0014388)
Supplement: Table S2 — (0.04 MB DOC) [file pone.0014388.s005.doc]

| **Primer number**   | **Primer sequence** |  | | --- | --- | | **33** | **TGCGCGCGCCA**TTTCTTGTTCCTTTCA | | **511** | CTACAACACGACCCTCTCCG | | **2511** | ACGCGTACACAACACACGGAC | | **2522** | **GCCCTGGAAGTACAGATTCTCGGTACCGCT**CAGGTCCTCCTCCG | | **3510** | **ATGGAGCAGAAGCTGATCTCGGAGGAGGACCTGGT**GAGCAAGGGCGAGGAGC | | **8581** | TGTGAAATTTGTGATGCTATTGC | | **8926** | **ACAAGGACGACGATGACAAA**ATGGTGAGCAAGGGCG | | **8927** | **CCCGCTACCTGCACTGGCACC**CACCATGGTTTCACTTAC | | **8928** | **CCCGCTACCTGCACTGGCACC**CAGGTCCTCCTCCGAGAT | | **8929** | **ACAAGGACGACGATGACAAA**TAATGAGCCCTCCTCCTCCTT | | **9152** | **CGAGATCAGCTTCTGCTCCAT**GGTTTCACTTACGTGT | | **9176** | **GCCAGTGCAGGTAGCGGGAGT**ATGTCCGACAGCGAGAAG | | **9177** | **TTGTCATCGTCGTCCTTGTAGTC**TTTCTTGGCTTTGGCAGA | |
| --- | --- | --- | --- | --- | --- | --- | --- | --- | --- | --- | --- | --- | --- | --- | --- | --- | --- | --- | --- | --- | --- | --- | --- | --- | --- | --- | --- | --- |
| ***Table S2****.* ***The******primers used for PCR amplification plasmid templates.*** *5’-overhang sequences used as complementary linkers for OE PCR are marked in bold.* |
