# Supplementary material for: Towards the Construction of Expressed Proteomes Using a Leishmania tarentolae Based Cell-Free Expression System
Source: PLoS One. 2010 Dec 21;5(12):e14388. doi: 10.1371/journal.pone.0014388 (PMC3006200; doi:10.1371/journal.pone.0014388)
Supplement: Table S3 — (0.05 MB DOC) [file pone.0014388.s006.doc]

| **Primer**   | **number** | **Gene ID** | **Sequence** | **Encoded protein** | | --- | --- | --- | --- | | **1** | **LmjF32.2030** | **CTGTACTTCCAGGGC**ATGTCGCAGAGCAGCTAC | **Rab2a** | | **2** | TGAAAACAGAGTGAGGCTG | | **3** | **LmjF32.0490** | **CTGTACTTCCAGGGC**ATGATTTATACCAGTGTCCCTT | **Rab4** | | **4** | TGTTCGCATGATCCGC | | **5** | **LmjF18.1130** | **CTGTACTTCCAGGGC**ATGAACACCCACCCACC | **Rab5** | | **6** | CGTGGCACTCAGCTGAT | | **7** | **LmjF10.1170** | **CTGTACTTCCAGGGC**ATGGAAGACTTGGTATACAAG | **Rab7** | | **8** | CACCTGTCGCAACATG | | **9** | **LmjF10.0910** | **CTGTACTTCCAGGGC**ATGGAGGAGACCAACCTC | **Rab11** | | **10** | CACAAGCAGCATGCAT | | **11** | **LmjF07.0560** | **CTGTACTTCCAGGGC**ATGAGCCACAAGTATATCTTC | **Rab14** | | **12** | CCACCTTTGCCCTCTCC | | **13** | **PFE0690c** | **CTGTACTTCCAGGGC**ATGACTGAGAATAGATCAAGAGA | **Rab1a** | | **14** | **GGCGCGCGCAA**TTAACAGGAACAAAAGGATTG | | **15** | **PFE0625w** | **CTGTACTTCCAGGGC**ATGAATGATAGCTATGATAGTTTATT | **Rab1b** | | **16** | **GGCGCGCGCAA**TCAACAACATTTCTTTTTGG | | **17** | **PFL1500w** | **CTGTACTTCCAGGGC**ATGTCTCCTTATGAATATTTGTTT | **Rab2** | | **18** | **GGCGCGCGCAA**TTAACAACAGCTAAATCCTG | | **19** | **MAL13P1.51** | **CTGTACTTCCAGGGC**ATGGGATGTTCATCAAGC | **Rab5b** | | **20** | **GGCGCGCGCAA**TCAAGGATTGTTATAATATAAAACTT | | **21** | **PFA0335w** | **CTGTACTTCCAGGGC**ATGGCTTATTATTTATCAAATTTA | **Rab5c** | | **22** | **GGCGCGCGCAA**TCAACAACATTTTTTTTTGG | | **23** | **PFI0155c** | **CTGTACTTCCAGGGC**ATGTCAAATAAAAAAAGAACCA | **Rab7** | | **24** | **GGCGCGCGCAA**TTAACAACAACGACTTTTGTAC | | **25** | **PF08_0110** | **CTGTACTTCCAGGGC**ATGAAAAATAAAAATAAGTATGATTATT | **Rab18** | | **26** | **GGCGCGCGCAA**TTAACAAGCGCAATTGG | |
| --- | --- | --- | --- | --- | --- | --- | --- | --- | --- | --- | --- | --- | --- | --- | --- | --- | --- | --- | --- | --- | --- | --- | --- | --- | --- | --- | --- | --- | --- | --- | --- | --- | --- | --- | --- | --- | --- | --- | --- | --- | --- | --- | --- | --- | --- | --- | --- | --- | --- | --- | --- | --- | --- | --- | --- | --- | --- | --- | --- | --- | --- | --- | --- | --- | --- | --- | --- | --- | --- | --- | --- | --- | --- | --- | --- | --- | --- | --- | --- | --- | --- | --- |
| ***Table S3****.* ***The******primers used for PCR amplification of* L.tarentolae *and* P.falciparum *Rab GTPases.*** *Primer sequences are numbered and annotated as in table 1. Gene ID are given according to EuPathDB (http://eupathdb.org).* |
