# Supplementary material for: Towards the Construction of Expressed Proteomes Using a Leishmania tarentolae Based Cell-Free Expression System
Source: PLoS One. 2010 Dec 21;5(12):e14388. doi: 10.1371/journal.pone.0014388 (PMC3006200; doi:10.1371/journal.pone.0014388)
Supplement: Table S4 — (0.03 MB DOC) [file pone.0014388.s007.doc]

| **Organism**   | **Gene Bank ID** | **Protein** |  | | --- | --- | --- | | ***Canis lupus*** | **404007** | **Rab7** | | ***Mus musculus*** | **19346** | **Rab6a** | | ***Mus musculus*** | **19331** | **Rab19** | | ***Mus musculus*** | **104886** | **Rab15** | |
| --- | --- | --- | --- | --- | --- | --- | --- | --- | --- | --- | --- | --- | --- | --- | --- |
| ***Table S4****.* ***The list of mammalian Rabs used in the study.*** |
